# Strategies for management of diabetes in the young in China

**DOI:** 10.1186/1687-9856-2015-S1-O17

**Published:** 2015-04-28

**Authors:** Xiaoping Luo

**Affiliations:** 1 Tongji Hospital, Wuhan, China

## 

The incidence of type 1 diabetes mellitus (T1DM) is rising rapidly in children, with an overall annual global increase of 3%. Approximately 79,100 children under age 15 develop T1DM annually. Although the reported incidence of T1DM in China was as low as 0.6 per 100,000 per annum in 1995, the number of children with T1DM cannot be underestimated within the large population. The number of type 2 diabetics in the young has been increasing significantly in the last decades.

To improve access to quality care for children with diabetes in China, the World Diabetes Foundation (WDF) planned a project to help to establish pediatric diabetes centers to increase the rate of diagnosis and to improve the quality of treatment for children with diabetes through training of health care professionals. A survey was conducted on the basis of pediatric diabetes centers across China to raise the awareness of pediatric diabetes, and to evaluate the diagnosis and treatment status of diabetes care in children. Multiple diabetes camps were organized for children and caregivers. The project commenced in December 2012 and will be completed in February 2015. Thirty three pediatric diabetes centers in 25 cities across China were established. A cross-sectional questionnaire survey about the children with diabetes was completed.

Until the end of December 2013, 1,224 children with T1DM were effectively enrolled into the program with a mean age of 7.28±3.75 (mean± SD). For the age distribution, 79% were in the range of 4-12 years old, of those 384 cases were younger than 5 and 840 cases were older than 5 years old. The male to female ratio was 46.8% to 53.2%. 49% of the patients presented with typical diabetic symptoms such as increased thirst, frequent urination, extreme hunger, weight loss and fatigue. 59.3% cases first presented with diabetic ketoacidosis (DKA). The overall incidence of hypoglycemia was 17.5% (20.3% in <5y vs 16.3% in ≥5y, P>0.05y). The total incidence of DKA was 59.3% (57.4% in <5y vs 60.2% in ≥5y, P>0.05). The average level of HbA1c was (11.1±3.2)%. Using HbA1c 7.5% as a cut-off, the incidence of hypoglycemia was 29.3% in the HbA1c below 7.5% group and 14.2% in the above 7.5% group (P<0.001). The incidence of DKA was 54% in the HbA1c below 7.5% group and 61% in the above 7.5% group (P>0.05). 97.2% of the patients received insulin treatment, while 83.3% with insulin injection and 14% adopted pump therapy. 78.8% of the patients were using multiple insulin injection regimes.

In China, T1DM is still the major form of diabetes among the young, although there is a tendency of increasing T2DM. Greater efforts need to be made to improve the awareness and quality of care. A task fprce on diabetes in children was recently formed by the Chinese Society of Pediatric Endocrinology and Metabolism (CSMEM) to facilitate nationwide research and collaboration.

